# Vitamin D Status and Physical Functioning in Nursing Home Residents after Improved Adherence to the Vitamin D and Calcium Recommendation—A Quasiexperimental Study

**DOI:** 10.1155/2024/2405429

**Published:** 2024-10-05

**Authors:** Charlotte Mortensen, Anne Marie Beck, Inge Tetens, Charlotte Jeppesen, Søren Frank Jørgensen, Leif Kofoed Nielsen, Michael Kristensen

**Affiliations:** ^1^ Department of Nursing and Nutrition Faculty of Health University College Copenhagen, Copenhagen, Denmark; ^2^ Dietetic and Nutritional Research Unit Herlev Gentofte Hospital, Hellerup, Denmark; ^3^ Department of Nutrition, Exercise and Sports Faculty of Science University of Copenhagen, Copenhagen, Denmark; ^4^ Department of Technology Faculty of Health University College Copenhagen, Copenhagen, Denmark

## Abstract

**Introduction:**

Dietary supplements with vitamin and calcium are recommended to nursing home residents in Denmark, but adherence to the recommendation is low. In a previous part of this study, we reported improved adherence by means of The Model for Improvement leading to increased awareness and change of workflows at two nursing homes. However, potential effects of this improved adherence are unknown.

**Objective:**

The objective of this substudy was to investigate if the improved adherence to the recommendation affected vitamin D status, muscle strength, and physical functioning of the residents.

**Methods:**

This was a 20-week quasiexperimental study involving 40 residents from two Danish nursing homes. Baseline and endpoint measurements took place in October 2021 and March 2022, respectively. Outcomes were number of residents taking vitamin D and calcium supplements; vitamin D status; handgrip strength; and physical functioning with timed-up-and-go test and 30-second chair stand test.

**Results:**

Prevalence of vitamin D supplement users increased from 45 to 78% (mean dose 41 *μ*g) and of calcium supplement users from 40 to 72% (mean dose 769 mg) (both *P*=0.002). Among those having blood sampled at both baseline and endpoint (*n* = 30), mean vitamin D status increased from 66.6 ± 31.7 nmol/L to 82.8 ± 26.3 nmol/L (*P* < 0.001), and more residents were vitamin D sufficient at endpoint (90 vs. 63%, *P*=0.021). Endpoint vitamin D status among supplement users was 88.2 ± 22.2 nmol/L, which was higher compared to nonsupplement users (55.3 ± 30.4 nmol/L, *P* < 0.01). No effects were seen on muscle strength or physical functioning.

**Conclusions:**

Increased supplementation with vitamin D using The Model for Improvement positively affected vitamin D status and prevalence of vitamin D sufficiency but did not affect muscle strength or physical functioning. Longer-term studies involving more residents are needed to investigate effects of improved adherence on these outcomes. This trial is registered with NCT04956705.

## 1. Introduction

Older adults residing at nursing homes are recommended a daily supplement of vitamin D, sometimes in combination with calcium, to support bone health and decrease risk of osteoporosis, falls, and fractures [1–4]. However, vitamin D deficiency, assessed as 25-hydroxyvitamin D (25(OH)D) < 30 nmol/L [5], is prevalent among nursing home residents where supplement use is low [3, 6–8], both because they spend limited time outside in the sun and because the capacity for vitamin D synthesis in the skin declines with age [9]. Moreover, appetite tends to decrease with age and few foods contain large amounts of vitamin D [8]. When it comes to calcium, the fractional absorption from the intestine decreases with age [10]. The above factors highlight the importance of following the recommendation of taking vitamin D and calcium as supplements, especially in countries with low food fortification and during winter at northern latitudes where vitamin D synthesis in the skin is negligible for 5 to 6 months a year [11]. However, we previously reported a low adherence to the recommendation of 20 *μ*g vitamin D and 800–1000 mg calcium supplements at Danish nursing homes [12]. In a previous quality improvement study with more than 100 nursing home residents, we targeted the barriers for giving the supplements using the Model for Improvement [13]. This resulted in a 102% improvement in the number of residents given the recommended supplements [14].

When it comes to Northern European countries, we only have few data on vitamin D status among nursing home residents. A recently published systematic review reported mean serum 25(OH)D concentration in the range of 34–40 nmol/L among Swedish nursing home residents from three different studies and around 30 nmol/L among nursing home residents in one Irish study [8]. No data were reported from Danish nursing homes. Moreover, to our knowledge, it has never been investigated to what extent vitamin D status may improve in this vulnerable group as a response to efforts of improving adherence to the supplement recommendation.

It has been suggested that vitamin D supplements can improve muscle strength of older adults [15], while other studies find no effects on muscle strength or physical functioning [16–19] or even adverse effects on these outcomes [20]. Thus, the importance of vitamin D supplementation for muscle function among nursing home residents is unclear.

Based on the above, the primary objectives of this study were to investigate whether an improved adherence to the vitamin D and calcium supplement recommendation in Danish nursing home residents affect their vitamin D status, physical functioning, and muscle strength.

## 2. Methods

### 2.1. Study Design

This was a 20-week winter trial with a quasi-experimental design. Baseline and endpoint measurements took place at two nursing homes in Denmark (55°N) in October 2021 and March 2022, respectively. The outcomes were the number of residents having daily supplements with vitamin D and calcium, their vitamin D status, physical functioning, and muscle strength.

This paper presents part two of the previously described study. It investigates the causal impact of improving implementation of the vitamin D and calcium supplement recommendation at the recruited nursing homes in a realistic setting. In part one, we conducted small-scale experiments under the Model for Improvement to improve adherence to the recommendation. The experiments were: (1) implementation of an information sheet about the recommendation to ensure improved supplement use among the residents and (2) adjustment of admission meetings to include the recommended supplements as a topic to address [14]. However, no outcomes related to effects of this improved adherence were included in that study.

### 2.2. Participants and Recruitment

The participants were all residents at the two nursing homes, as described in our previous paper [14]. Inclusion criteria were the ability to understand and speak Danish to understand the study procedures and to give informed consent. Exclusion criteria were receiving medication, which may induce adverse effects in combination with vitamin D and/or calcium supplements, known kidney disease, being terminally ill, or being dependent on a guardian. Before initiating the recruitment process the nursing home staff provided the project responsible with names of residents who were not to be contacted based on the above criteria. The project responsible had individual information meetings with the remaining residents in their apartments at the nursing homes and informed them about the study's aim, design, methods, importance, and potential risks. Moreover, they received written information on the study. If a resident wished to take time to consider their participation or talk with their relatives before deciding, the project responsible followed up after 2-3 days.

### 2.3. Measurements

#### 2.3.1. Vitamin D and Calcium Supplement Intake

We assessed a resident's intake of vitamin D and calcium supplements and multivitamins by review of patient records. Few residents purchased supplements by themselves (with no prescription from the general practitioner). In these cases, the project responsible had information on doses, etc., directly from the resident. For more details, see the paper on part one of the study [14].

#### 2.3.2. Hydroxyvitamin D Measurements

A venous blood sample was drawn from the residents at baseline and endpoint, respectively. Sampling took place from 9 to 12 am after a self-selected breakfast. The venous blood samples were drawn by trained staff and according to guidelines in serum tubes with gel (VACUETTE® TUBE 3.5 ml CAT Serum Separator Clot Activator (Greiner Bio-One, Kremsmünster, Austria)) and left for coagulation. Within six hours after collection, samples were centrifuged at 2000 × g for 10 minutes. The serum was transferred to cryotubes (2 mL Redline, Heinz Herenz Hamburg, Germany) and stored at −80°C until analysis. All samples were anonymized using an ID-log connecting the study participants with their biological material. The serum samples were analyzed as batch analyses for vitamin D status assessed as 25-hydroxyvitamin D (*D*_3_+*D*_2_), using the competitive chemiluminescence binding assay at a Liaison XL (DiaSorin Inc., Italy) [21]. Both baseline and endpoint samples were analyzed after the endpoint samples were collected. After analysis, the remaining sample material was destructed. Vitamin D status categories used were according to Institute of Medicine (IOM) and the Nordic Nutrition Recommendations as follows: Vitamin D deficiency defined as serum 25(OH)D < 30 nmol/L and vitamin D sufficiency defined as ≥ 50 nmol/L [5, 22].

#### 2.3.3. Muscle Strength

We measured muscle strength as handgrip strength (HGS) using a hydraulic Jamar hand dynamometer (Patterson Medical, UK), which is the most widely used instrument [23]. HGS is considered representative of whole-body strength and a predictor of physical functioning, hospital length of stay, and mortality [24]. Moreover, it has been reported to have a high test-retest reliability in older adults [25]. The participants sat on a chair with back- and armrests or in their wheelchair. Elbow was in a 90° flexion and the arm placed on the armrest. The dynamometer was gently supported below by the instructor to minimize the effect of gravity as suggested by others [23]. The residents performed three measurements with the dominant hand and with a 60-second break between measurements. Maximum HGS was the highest value (to the nearest 1 kg) of the three measurements. Roberts et al. previously suggested the highest value of six measurements to be the maximum muscle strength (three with each hand) [23], but we modified this approach with the target group in mind to avoid fatigue. A HGS of <16 kg for women and <27 kg for men, respectively, has been suggested as sarcopenia cutoffs [26].

#### 2.3.4. Physical Functioning

Physical functioning was estimated with the timed-up-and go test and the 30-s chair-stand test. The timed-up-and-go test (TUG test) is a validated and recommended tool to evaluate fall risk among older adults [27, 28]. Participants sat in a chair with back- and armrests. They were instructed to rise from seated position, walk at a self-selected and safe pace to a line on the floor 3 m from the front of the chair, turn around, walk back, and sit down. Time was started at the signal “go” and was stopped when the participant sat on the chair again. If the participant normally used a walker, they also used it during the test [29]. The TUG test was performed three times with a 120-second break between tests. The test with the best time was used as the final score of the participant. Previously, it has been suggested that a score of more than 12 seconds in the TUG test indicates increased fall risk [27].

The 30-second chair-stand test (30CST) has a high test-retest reliability among older adults and is a valid measure of lower body strength [30] and fall risk [31] by counting how many full stands can be achieved in 30 seconds. The participants sat in a chair with back- and armrests. We used the same chair at both baseline and endpoint to ensure identical height of the chair at both times. The instructor demonstrated the test, and participants were instructed to use the armrests while standing to ensure a safe test. Thus, this is a modified version of the standard test, which is without armrests [30]. An instructor stood behind for safety, made a countdown, and started a stopwatch at the signal “go.” Time was started even if the participant did not initiate the process of standing immediately. The instructor silently counted the number of full stands within the 30-second time limit. If the last stand was more than halfway up, this counted as a full stand. According to the guidelines, decreased physical functioning is prevalent if a resident performs less than nine stands in a standard test with no armrests, and it is recommended to perform an additional test of gait speed if a resident performs from five to eight stands [32].

### 2.4. Statistical Analyses

Data were analyzed with the statistical software IBM SPSS Statistics 29.0.1.0. Descriptive data are presented as *n* (%) or mean and SD when appropriate. We checked variables for normality with Shapiro–Wilk test and visually with Q-Q plots. Mean changes in continuous outcome variables (vitamin D status, physical functioning, and muscle strength) from baseline to endpoint were examined with paired *t*-tests. Corresponding changes in categorical variables (supplementation Y/N, vitamin D status category) were examined with McNemar's test. Comparisons in vitamin D status, physical functioning, and muscle strength at endpoint between vitamin D supplement users and nonusers were done with independent samples *t*-tests.

It has previously been suggested that effects of vitamin D status on soft tissues may require a serum 25(OH)D > 75 nmol/L [5, 33]. Moreover, The European Calcified Tissue Society defines sufficiency as a serum 25(OH)D > 75 nmol/L [34]. Based on this, we stratified changes from baseline to endpoint in the physical tests, and muscle strength test by vitamin D status at endpoint (either <75 nmol/L or ≥75 nmol/L) and compared groups with independent samples *t*-test. Associations between categorical variables (supplementation Y/N and vitamin D status category) were tested with chi-squared test or Fisher's Exact Test when more than 20% of cells had expected frequencies <5. A *P* value below 0.05 was considered significant.

### 2.5. Power Calculation

The power calculation was based on the wish to show a significant change in vitamin D status after 20 weeks. A previous Danish study showed a mean vitamin D status of 35 ± 19 nmol/L among more than 9,600 community-dwelling older adults [35]. As the older adults in the present project are nursing home residents, we expected a lower initial vitamin D status. Assuming a baseline vitamin D status of 27 nmol/L (delta = 8), a power of 0.8, and a significance level of 0.05, this gave a required sample size of *n* = 90. Taking missing blood samples into account, a total of *n* = 100 residents would preferably be recruited.

## 3. Results

### 3.1. Participant Flow and Timeline

**Figure 1:**
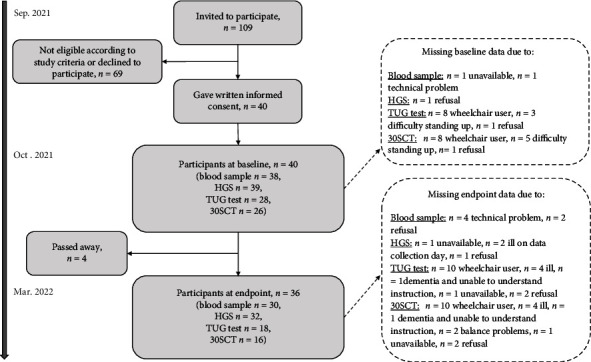
Study participant flow and number of participants with data on blood sampling, handgrip strength, timed-up-and-go test, and chair-stand test at baseline and endpoint, respectively. Explanations for missing data are shown in dotted boxes at the right. HGS: hand grip strength; TUG test: timed-up-and-go test; 30SCT: 30-second chair-stand test.

The timeline of the study and the flow of the study participants are shown in Figure 1.

### 3.2. Baseline Characteristics

As seen in Figure 1, a total of 40 residents (37%), all Caucasians, at the two nursing homes gave written informed consent.  Table 1 shows baseline characteristics of the participants. Frequencies of deficient (<30 nmol/L), insufficient (30–49.9 nmol/L), and sufficient (≥50 nmol/L) vitamin D status were 21%, 16%, and 63% of residents, respectively. Having vitamin D supplements was more prevalent than having calcium supplements and less than half of the residents had the recommended supplements. The dose of vitamin D most often consumed, that is., 38 *μ*d/day, was nearly twice as large as the officially recommended dose of 20 *μ*g/day.

Mean duration of the TUG test was 27 seconds which is well above the cutoff of >12 seconds indicating increased fall risk [27]. Residents had a mean of seven stands in the 30CST, which is somewhat lower than older adults (>80 years) from the Copenhagen Sarcopenia Study in which mean number of stands where 12 and 13 in women and men, respectively [36]. In addition, our data indicate decreased physical functioning according to Danish guidelines recommending additional test of gait speed if a resident performs five to eight stands [32]. Mean HGS for women and men, respectively, were 15.5 kg and 27.2 kg. These values are somewhat comparable to grip strength among similar age groups reported in a previous meta-analysis [37] but lower compared to older Danish adults (>80 years) in the Copenhagen Sarcopenia Study in which mean HGS was 20.3 kg and 33.7 kg in women and men, respectively.

### 3.3. Changes in Vitamin D and Calcium Supplement Intake

Four residents passed away before endpoint measurements, and frequency of residents having the supplements are therefore based on 40 and 36 residents at baseline and endpoint, respectively. Among the four deceased, two had vitamin D and calcium supplements at baseline. The prevalence of residents having vitamin D supplements in any dose increased from 45% at baseline to 78% at endpoint, whereas the prevalence of having calcium supplements increased from 40% to 72% (both *P*=0.002).

Among the vitamin D users, the mean dose of vitamin D at baseline was 37.3 *μ*g/day. At endpoint, mean vitamin D intake from supplements was 41.1 *μ*g/day. The most frequently consumed dose of vitamin D was 38 *μ*g/day at both baseline and endpoint (as this corresponded to two tablets of 19 *μ*g). Among the calcium supplement users, the mean baseline dose was 750 mg/day, which increased to 769.2 mg/day at endpoint. The most frequently consumed dose was 800 mg/day at both baseline and endpoint.

### 3.4. Effects on Vitamin D Status

**Figure 2:**
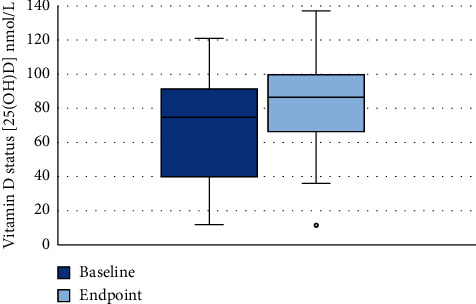
Boxplot of vitamin D status assessed as 25(OH)D at baseline and endpoint (*n* = 30). The blue boxes illustrate the range between lower and upper quartiles (interquartile range), the black line is median, and whiskers are minimum and maximum values. One outlier is shown with a circle. 25(OH)D: 25-hydroxyvitamin D.

A total of 30 participants had blood drawn at both baseline and endpoint. Mean baseline vitamin D status among these was 66.6 ± 31.7 nmol/L, and this increased to 82.8 ± 26.3 nmol/L (*P* < 0.001, Figure 2), corresponding to a 24% increase in serum 25(OH)D.

Among the 30 participants having blood sampled at both times, the prevalence of sufficiency (25(OH)D ≥ 50 nmol/L) increased during the intervention from 63% (*n* = 19) at baseline to 90% (*n* = 27) at endpoint (*P*=0.021). The prevalence of vitamin D deficiency (25(OH)D < 30 nmol/L) changed from 17% (*n* = 5) to 3% (*n* = 1) at endpoint, although this was insignificant (*P*=0.125).

Vitamin D status among residents receiving vitamin D supplements at endpoint was 88.2 ± 22.2 nmol/L, whereas vitamin D status among those not having vitamin D supplements at endpoint was 55.3 ± 30.4 nmol/L (Table 2) which is a difference between groups of 32.9 nmol/L (*P* < 0.01).

### 3.5. Effects on Physical Functioning and Muscle Strength

At baseline, the number of residents conducting the HGS test, TUG test, and the 30SCT, were 39, 28, and 26, respectively. At endpoint, corresponding numbers among the 36 remaining participants were 32, 18, and 16, respectively. Reasons for not performing the tests are shown in Figure 1. Despite improved mean vitamin D status, mean HGS, TUG test, or 30SCT did not change from baseline to endpoint (*P*=0.67, 0.16, and 0.66, respectively).

### 3.6. Effects According to Vitamin D Supplement Intake

When comparing vitamin D supplement users (*n* = 28) with nonusers (*n* = 8) at endpoint, the performance of mean HGS, TUG test, or 30SCT did not differ between groups (Table 2).

### 3.7. Effects According to Vitamin D Status Category

When comparing changes from baseline to endpoint in the physical tests and muscle strength stratified by vitamin D status, either <75 nmol/L or ≥75 nmol/L, no differences were seen between groups (*P*=0.34, 0.25, and 0.55, respectively).

### 3.8. Associations between Vitamin D Supplement Intake and Vitamin D Status Category

We found a significant association between taking vitamin D supplements and vitamin D status category at baseline. That is, at baseline, all residents consuming vitamin D were vitamin D sufficient (serum 25(OH)D ≥ 50 nmol/L), whereas 67% of residents not consuming vitamin D at baseline were vitamin D insufficient (*P*=0.001). In addition, at endpoint, we saw a tendency to an association, i.e., 96% of the residents consuming vitamin D were vitamin D sufficient, whereas 40% of residents not consuming vitamin D where vitamin D insufficient (*P*=0.064).

## 4. Discussion

In this quasi-experimental study, we found increased mean vitamin D status after a 20-week winter period among the residents living at two nursing homes at which the Model for Improvement was used to elicit a better adherence to the official recommendation of supplementation with vitamin D and calcium. Correspondingly, only 10% of the residents were vitamin D insufficient (25(OH)D < 50 nmol/L) at endpoint in March. Vitamin D status was increased by 21 nmol/L among supplement users and was reduced by 11 nmol/L among nonsupplement users at endpoint. However, despite improved 25(OH)D concentration during winter, neither increased nor reduced mean muscle strength or physical functioning was seen.

To our knowledge, only one study has been performed with a similar design and with vitamin D status as outcome. In an American study in which the general practitioners of the nursing home residents were urged to increase the number of vitamin D prescriptions through an educational letter, vitamin D status was determined in a subgroup of 11 residents before and after the 6-month intervention period. Although the mean changes in vitamin D status were not reported, it was noted that only two (i.e., 18%) [38] had endpoint 25(OH)D > 75 nmol/L. In comparison, 63% had endpoint vitamin D status >75 nmol/L in the present study. In this regard, it should be noted that the mean consumed vitamin D dose in our study was higher than the officially recommended dose of 20 *μ*g/day, which may explain that a relatively high number of the residents had endpoint 25(OH)D > 75 nmol/L. Therefore, even though our results show that supplementation with vitamin D effectively increases vitamin D status in this vulnerable group, we cannot know to what extent serum 25(OH)D concentration would increase if only the recommended doses were prescribed.

The mean improvement of vitamin D status at the two included nursing homes corresponded to 24%. A previous 1-year longitudinal study including both teenage girls and older Danish women (*n* = 106) estimated that vitamin D status from summer to winter improves with an average of 41% if initiating taking vitamin D supplements during winter [39]. As baseline mean vitamin D status was >50 nmol/L in the present study, this may lower the increase in vitamin D status. When looking at the nonsupplement users at endpoint, these had a vitamin D status of 55 nmol/L in March. This is somewhat higher than expected from other studies. A study obtaining blood samples from 545 Swedish nursing home residents from January to March showed a mean 25(OH)D of 34 nmol/L and insufficiency prevalence of 82%. Only 17% of these residents had vitamin D supplements [40]. A Danish study including seasonal blood samples from more than 3,000 persons (2–69 years) showed that serum 25(OH)D was 36 nmol/L and 43 nmol/L among male and female adult nonsupplement users, respectively. The corresponding serum 25(OH)D for supplement users was 63 nmol/L and 72 nmol/L, respectively [41]. Endpoint serum 25(OH)D for supplement users in the present study was 88 nmol/L which again may reflect the higher doses prescribed.

We found no effects on muscle strength or physical functioning from vitamin D and calcium supplementation. Several factors may contribute to this. First, we included a relatively small sample size, and among the recruited participants, we saw that fewer participants were able to have blood sampled and perform the physical tests at endpoint compared to baseline. Therefore, we must consider the likelihood of type 2 errors. Second, baseline vitamin D status was higher than expected with a mean serum 25(OH)D of 65 nmol/L, which may reflect vitamin D synthesis from the preceding summer months, and the fact that 45% were vitamin D supplement users at baseline. As suggested by Byers et al., a beneficial effect on muscle strength and physical performance may not be seen when vitamin D status is increased from an already adequate state [42], so this may explain that we did not see an effect on muscle outcomes. This is in accordance with a recently published study concluding that vitamin D-replete individuals do not benefit from vitamin D supplementation [43]. Third, the study design may contribute to the difficulty observing an effect on muscle strength and physical function. As our study was an effectiveness trial, we wished to measure the degree of beneficial effect from improved vitamin D supplement use in a realistic setting rather than studying effects under controlled circumstances as in an efficacy trial where the preferred design would be a placebo-controlled randomized trial [44].

Even though we did not see an effect of vitamin D supplementation and/or improved vitamin D status on physical functioning, it could be speculated that improved 25(OH)D during winter may decelerate a decline in physical activity level. A recent American study among low-functioning older adults (*n* = 571, 70+ years) reported an increased decline in physical activity level after up to 2 years of follow-up if baseline serum 25(OHD) was <50 nmol/L compared to ≥ 50 nmol/L [45]. Therefore, ensuring that nursing home residents do not become vitamin D insufficient may be important for overall mobility and prevention of decreased physical function over time, and our finding of status quo in muscle strength and physical function after a 20-week winter period may be viewed as a positive result.

Limitations of the present study include the small sample size included and thereby lack of power as only 40 residents (37%) volunteered to participate. One contributing factor may be that data collection took place during the COVID-19 epidemic. In addition, due to the study design, there was no randomization. Therefore, we cannot know if residents taking vitamin D and calcium supplements differed in other aspects. For instance, it is plausible that residents following the recommendation also have more appetite and thus get more vitamin D from the diet. Moreover, they may be more physically active, spend more time outside, and thereby have increased cutaneous vitamin D synthesis. However, even though we did not estimate dietary vitamin D intake, physical activity level, or outdoor time, we expect variations between residents to be relatively small and of low influence on vitamin D status as compared to supplementation. Another limitation of the present study is that we assumed that data on supplement intake in patient records were equal to consumed doses, since it was not realistic to observe actual consumption. However, according to the health care professionals, supplements appearing in patient records were always dosed and consumption checked regularly.

Strengths of the present study include the use of standardized and relevant methods with a high test-retest reliability to estimate muscle strength and physical functioning in the older adults. Moreover, tests were performed at approximately the same time of the day at baseline and endpoint to avoid influence of diurnal variations in the energy level. Also, blood samples were drawn at the same time of the day at baseline and endpoint. 25-hydroxyvitamin D measurements were performed as part of the diagnostic routine and in accordance with guidelines from the Vitamin D Standardization Program (VDSP) using standard reference material (National institute of Standardization and Technology, NIST), thereby allowing comparison of analyte levels to results published using the reference method liquid chromatography–tandem mass spectrometry (LC-MS/MS) [21].

## 5. Conclusions

In conclusion, supplementation with vitamin D is practical and effective to improve vitamin D status and prevalence of vitamin D sufficiency in nursing home residents during the winter season. Even though physical functioning and muscle strength were not affected in this study, following the official recommendation of taking supplements with vitamin D and calcium should still be urged based on the evidence of effects on bone health.

## Figures and Tables

**Table 1:** Characteristics of the 40 participants at baseline.

Variable	

Women/men, *n* (%)	27 (68)/13 (32)
Age, years, mean ± SD (min, max)	80.9 ± 8.8 (58.5, 94.3)
Supplementation	
Vitamin D supplement user, *n* (%)	18 (45)
Mean vitamin D dose among users, *μ*g/day, mean ± SD	37.3 ± 7.7
Frequency of vitamin D doses among users	
19 *μ*g/d, *n* (%)	2 (11)
38 *μ*g/d, *n* (%)	13 (72)
40 *μ*g/d, *n* (%)	1 (6)
50 *μ*g/d, *n* (%)	2 (11)
Calcium supplement user, *n* (%)	16 (40)
Mean calcium dose among users, mg/day, mean ± SD	750 ± 137
Frequency of calcium dose among users	
400 mg/day, *n* (%)	2 (12)
800 mg/day, *n* (%)	14 (88)
Multivitamin user, *n* (%)	10 (25)
Blood measurements^a^	
Vitamin D status, nmol/L, mean ± SD (min, max)	65.2 ± 32.3 (12, 121)
Vitamin D deficiency (25(OH)D < 30 nmol/L), *n* (%)	8 (21)
Vitamin D sufficiency (25(OH)D ≥ 50 nmol/L), *n* (%)	24 (63)
Physical tests	
HGS (kg), mean ± SD (min, max)^b^	19.4 ± 10.8 (7, 58)
TUG test (*s*) mean ± SD (min, max)^c^	27.5 ± 23.9 (7.2, 140.1)
30CST (no.) mean ± SD (min, max)^d^	7.3 ± 2.6 (2, 12)

Data available for ^a^*n* = 38, ^b^*n* = 39, ^c^*n* = 28, and ^d^*n* = 26 participants. 25(OH)D: 25-hydroxyvitamin D; HGS: handgrip strength; TUG test: timed-up-and-go test; 30CST: 30-second chair-stand test.

**Table 2:** Endpoint values and change from baseline to endpoint for vitamin D status, muscle strength, and physical functioning stratified by use or nonuse of vitamin D at endpoint.

	Vitamin D at endpoint^a^	No vitamin D at endpoint^b^	*P* value
Vitamin D status (nmol/L), mean ± SD			
Endpoint	88.2 ± 22.2	55.3 ± 30.4	<0.01^∗^
Change	21.7 ± 26.6	−11.3 ± 9.2	0.01^∗^
HGS (kg), mean ± SD			
Endpoint	18.7 ± 9.3	23.3 ± 10.3	0.27
Change	−0.2 ± 5.8	2.3 ± 3.5	0.27
TUG test (*s*) mean ± SD			
Endpoint	26.0 ± 10.6	19.3 ± 7.3	0.19
Change	4.0 ± 7.5	−0.2 ± 5.4	0.25
30SCT (no.) mean ± SD			
Endpoint	7.8 ± 2.4	7.0 ± 1.8	0.50
Change	−0.3 ± 1.5	1.0 ± 1.8	0.14

^∗^Significant difference between groups. ^a^*n* = 28 and ^b^*n* = 8. HGS: handgrip strength; TUG test: timed-up-and-go test; 30CST: 30-second chair-stand test.

## Data Availability

The data that support the findings of this study are available from the corresponding author upon reasonable request.

## Abbreviations

25(OH)D: 25-Hydroxyvitamin D
30SCT: 30-Second chair-stand test
HGS: Handgrip strength
IOM: Institute of medicine
LC-MS/MS: Liquid chromatography–tandem mass spectrometry
TUG test: Timed-up-and-go test
